# COVID-19 en pacientes en hemodiálisis en Colombia: reporte de siete casos

**DOI:** 10.7705/biomedica.5743

**Published:** 2020-11-12

**Authors:** Gustavo Aroca, María Vélez-Verbel, Andrés Cadena, Lil Geraldine Avendaño, Sandra Hernández, Angélica Sierra, Omar Cabarcas, Santos Ángel Depine

**Affiliations:** 1 Facultad de Nefrología, Universidad Simón Bolívar, Barranquilla, Colombia Universidad Simón Bolívar Facultad de Nefrología Universidad Simón Bolívar Barranquilla Colombia; 2 Departamento de Nefrología, Clínica de la Costa, Barranquilla, Colombia Departamento de Nefrología Clínica de la Costa Barranquilla Colombia; 3 Facultad de Ciencias de la Salud, Programa de Medicina, Universidad Libre, Barranquilla, Colombia Universidad Libre Facultad de Ciencias de la Salud Universidad Libre Barranquilla Colombia; 4 Confederación de Asociaciones de Diálisis de la República Argentina, Capital Federal, Argentina Confederación de Asociaciones de Diálisis de la República Argentina Capital Federal Argentina

**Keywords:** infecciones por coronavirus, síndrome respiratorio agudo grave, diálisis renal, informes de casos, Coronavirus infections, severe acute respiratory syndrome, renal dialysis, case reports

## Abstract

A finales del 2019 se inició en Wuhan, China, el brote de un nuevo coronavirus que se dispersó por todo el mundo infectando y cobrando miles de vidas. Se ha encontrado que ciertas comorbilidades constituyen factores de riesgo para resultados poco satisfactorios de la enfermedad, pero es poco lo que se ha descrito sobre pacientes en hemodiálisis, a pesar de tratarse de una población de alto riesgo de infección, complicaciones y muerte. En este artículo se describe el curso clínico, las manifestaciones clínicas y las complicaciones de la COVID-19 en siete pacientes en hemodiálisis permanente y se hacen recomendaciones para el manejo de pacientes con enfermedad renal crónica.

## Abstract (en)

At the end of 2019, in Wuhan, China, the outbreak of a new coronavirus began and quickly spread throughout the world infecting and claiming thousands of lives. To date, certain comorbidities are known to be risk factors for unsatisfactory disease outcomes, but little has been reported regarding hemodialysis patients despite being a population at high risk of infection, complications, and death.

Here we describe the clinical course, clinical manifestations and complications of COVID-19 in seven patients on permanent hemodialysis. We also make recommendations for the management of patients with chronic kidney disease.

A finales del 2019 se inició en Wuhan, China, el brote de un nuevo coronavirus que infectó a más de 70.000 individuos y cobró más de 1.800 vidas durante los primeros cincuenta días de actividad ^1^. El 11 de marzo de 2020 la Organización Mundial de la Salud (OMS) declaró la pandemia, hoy presente en más de 109 países, con 11'327.790 casos y 532.340 muertes registradas hasta el 6 de julio de 2020 ^2^. Hasta el 6 de julio de 2020 se habían reportado en Colombia 120.281 casos y 4.210 muertes, con 146 nuevos fallecimientos en esa fecha, 57 de ellos en Barranquilla y el departamento del Atlántico, donde habían muerto más de 1.000 personas ^3^^,^^4^.

El virus fue denominado SARS-CoV-2 y la enfermedad COVID-19 por el Comité Internacional de Taxonomía de Virus ^5^. La principal forma de presentación de la enfermedad es la neumonía, la cual se manifiesta con fiebre, tos, disnea, dolor de garganta, mialgias, trastornos gastrointestinales y rinorrea ^6^, y las complicaciones más frecuentes son el síndrome de dificultad respiratoria aguda con requerimiento de respiración mecánica asistida, insuficiencia renal aguda, arritmias, infarto agudo del miocardio y choque ^7^. Se ha determinado, además, que puede afectar otros tejidos diferentes al pulmonar, como el corazón, las vías digestivas, los riñones, la sangre y el sistema nervioso ^8^.

Se han encontrado múltiples factores de mal pronóstico asociados con el desarrollo de complicaciones, entre ellos, ciertos biomarcadores como la linfopenia, la elevación de los niveles de las enzimas hepáticas, la lactato deshidrogenasa (LDH), los reactantes de fase aguda del tipo de la proteína C reactiva y la ferritina, el dímero-D, las troponinas y la creatina cinasa, así como el tiempo de coagulación prolongado. También se han considerado factores de riesgo la edad avanzada y la presencia de enfermedad cardiovascular, diabetes mellitus, hipertensión, enfermedad pulmonar crónica, cáncer y enfermedad renal crónica ^9^^-^^11^.

Con corte al 31 de diciembre del 2019 en Colombia se registraba una prevalencia de 925.996 casos de pacientes con enfermedad renal crónica en cualquiera de sus estadios; 45.615 se encontraban en estadio 5 y de estos, 43.153 estaban en tratamiento de reemplazo renal, 59 % en hemodiálisis, 22 % en diálisis peritoneal y el 18 % había recibido trasplante ^12^.

Los pacientes con enfermedad renal crónica en estadio 5 dependientes de hemodiálisis no pueden cumplir la cuarentena impuesta por la pandemia de COVID-19, ya que deben asistir tres veces por semana a diálisis. Se trata de pacientes que sufren inmunosupresión y desnutrición, además de las condiciones de hipertensión arterial y diabetes mellitus, principales causas de la enfermedad renal crónica, todo lo cual contribuye a un mayor riesgo de gravedad de la COVID-19 ^13^^,^^14^.

La información sobre la epidemiologia de la COVID-19 en pacientes en diálisis permanente es limitada y controversial, a pesar de tratarse de pacientes de alto riesgo debido a sus múltiples comorbilidades. A ello se suman factores logísticos que deben atenderse dada su obligada y recurrente asistencia a centros de salud y la proximidad física con otros pacientes durante la diálisis por la gran demanda: la norma habitual establece una superficie de 6 m^2^ por unidad de diálisis, con una separación entre ellas de 60 cm a 1 metro. En muchos casos también deben considerarse las condiciones de vida de extrema pobreza y la poca alfabetización de los pacientes, lo que dificulta aún más el aislamiento y el distanciamiento social.

En China no se reportaron diferencias en el resultado final de la COVID-19 entre estos pacientes y la población general, sin embargo, en Italia y Estados Unidos se describió un mayor ingreso a las unidades de cuidados intensivos ^15^^,^^16^. En este artículo se describen siete casos de pacientes en diálisis permanente con COVID-19 en un centro hospitalario de cuarto nivel de Barranquilla, Colombia.

## Reporte de casos

### Caso 1

Hombre de 43 años residente en Barranquilla (Atlántico) con enfermedad renal crónica permanente secundaria a glomerulonefritis no especificada e hipertensión, en tratamiento de hemodiálisis desde el 19 de junio de 2019 con cuatro horas por sesión tres veces a la semana mediante fístula arteriovenosa con diuresis residual. La COVID-19 inició el 6 de junio de 2020 con tos acompañada de expectoración de tonalidad verdosa, anorexia, anosmia, ageusia, astenia y adinamia. Negó contacto con personas diagnosticadas con COVID-19. Desde el inicio de los síntomas el paciente no acudió a su centro de diálisis para continuar su terapia.

**Figura 1:**
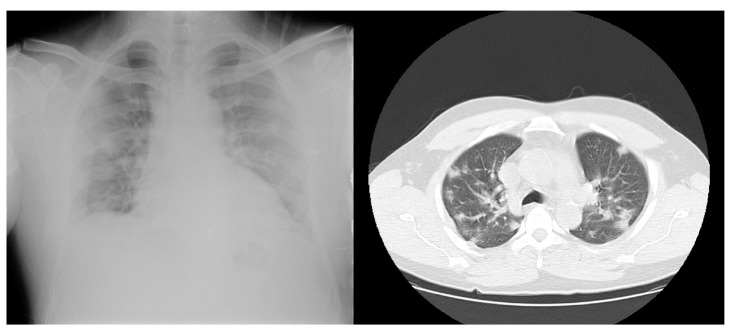
Radiografía y tomografia computarizada de tórax en el caso 1

En el examen físico de ingreso se encontró presión arterial de 138/80 mm Hg, frecuencia cardiaca de 100 latidos por minuto, temperatura de 39,3 °C, frecuencia respiratoria de 24 respiraciones por minuto, saturación de oxígeno del 94 %, examen pulmonar con disminución del murmullo vesicular y estertores crepitantes bilaterales subescapulares; no se evidenciaron edemas, asterixis, deterioro del estado de consciencia ni debilidad muscular. Los resultados de los exámenes de laboratorio se presentan en el cuadro 1; la radiografía de tórax reveló infiltrados intersticiales y patrón hiliar de compromiso mixto y la tomografía de tórax, infiltrados bilaterales con patrón de vidrio esmerilado a nivel periférico y central en ambos campos pulmonares (figura 1).

**Cuadro 1:** Exámenes de laboratorio durante la hospitalización en el caso 1

Examen de laboratorio	Día 1	Día 2	Día 4	Día 6	Día 9
Leucocitos (número por mm^3^)	3.600	NR	2.600	3.800	7.800
Neutrófilos (número por mm^3^)	2.500	NR	1.800	2.500	6.300
Linfocitos (número por mm^3^)	700	NR	400	900	800
Hemoglobina (g/dl)	9,2	NR	8,8	8,7	7,5
Plaquetas (número por mm^3^)	87.000	NR	117.000	133.000	223.000
Velocidad de sedimentación (mm/h)	30	NR	NR	NR	NR
Tiempo de trombina (s)	10,3	NR	11	NR	NR
Tiempo parcial de tromboplastina (s)	24,4	NR	39	NR	NR
INR	0,9	NR	1	NR	NR
Creatinina (mg/dl)	21,6	NR	18	18,5	21,1
BUN (mg/dl)	88	NR	43	61	86
Urea (mg/dl)	187	NR	92	130	184
Sodio (mEq/L)	134	NR	145	143	135
Potasio (mEq/L)	5,1	NR	4,8	5,1	6,1
Magnesio (mg/dl)	2,2	NR	NR	NR	NR
Calcio (mg/dl)	9,3	NR	8,7	NR	6,4
Cloro (mEq/L)	93	NR	108	106	98
GOT (UI/L)	52	NR	127	NR	199
GPT (UI/L)	63	NR	126	NR	206
Bilirrubina total (mg/dl)	0,6	NR	NR	NR	NR
Bilirrubina directa (mg/dl)	0	NR	NR	NR	NR
Bilirrubina indirecta (mg/dl)	0	NR	NR	NR	NR
Fosfatasa alcalina (Ui/L)	62	NR	NR	NR	NR
Deshidrogenasa láctica (U/L)	268	NR	408	NR	929
PCR (mg/dl)	NR	4,1	NR	NR	23,6
Panel respiratorio: influenza A, influenza B y H1N1 2009	NR	Negativo	NR	NR	NR
Parcial de orina	NR	NR	NR	Color: amarillo	NR
				Aspecto: ligeramente turbio	
				pH: 6,5	
				Densidad: 1.015	
				Proteínas: 1.000 mg/dl	
				Sangre: +	
				Leucocitos: negativo	
				Nitritos: negativo	
				Glucosa: 100 mg/dl	
				Cuerpos cetónicos: negativo	
				Urobilinógeno: normal	
				Bilirrubina: negativo	
				Células epiteliales altas:	
				0-2 por campo, bacterias:	
				escasas, leucocitos: 0-2 por	
				campo, hematíes 2-4 por	
				campo.	
Hemocultivo	NR	Negativo	NR	Negativo	NR
Urocultivo	NR	Negativo	NR	Negativo	NR
Coprocultivo	NR	NR	NR	Negativo	NR
Detección de gen regulador de toxinas para *Clostridium difficile*	NR	NR	NR	Negativo	NR

NR: no reportado: INR: *International Normalized Ratio;* BUN: *Blood Urea Nitrogen;* GOT: *Glutamic-Oxaloacetic Transaminase;* GPT: *Glutamic-Pyruvic Transaminase;* LDH: *Lactate Dehydrogenase*

El paciente fue hospitalizado en aislamiento respiratorio; se reinició la hemodiálisis en días alternados con sesiones de cuatro horas, ultrafiltración de 2.000 ml, filtro de 170, bomba de sangre a 300 ml/min, y flujo del líquido dializado de 500 ml/min. Se le administró tratamiento antibiótico con piperacilinatazobactam en dosis de 2,25 g intravenosos cada 12 horas y vancomicina en dosis de 1 g cada 48 horas después de la diálisis, así como 3 l/min de oxígeno suplementario por cánula nasal. La muestra de hisopado nasofaríngeo fue positiva para SARS-CoV-2. Durante la hospitalización presentó disnea de pequeños esfuerzos, náuseas, vómitos y deposiciones diarreicas, por lo que se le adicionaron 500 mg de metronidazol cada 8 horas por vía oral. La evolución clínica fue satisfactoria y hubo mejoría de la dinámica respiratoria, por lo que se dio el alta médica el día décimo de internación, sin complicaciones, indicándose continuidad de tratamiento dialítico en su centro de referencia.

### Caso 2

Mujer de 43 años, residente en Barranquilla (Atlántico) con enfermedad renal crónica permanente e hipertensión, en tratamiento de hemodiálisis tres veces a la semana en sesiones de cuatro horas desde noviembre de 2019 mediante catéter "tunelizado" (sic) tras la utilización fallida de dos fístulas arteriovenosas, y sin diuresis residual. La COVID-19 inició el 8 de junio de 2020 con picos febriles no cuantificados acompañados de episodios diarreicos (4 deposiciones diarias) y disnea leve. Negó contacto con personas con COVID-19. Había dejado de asistir a su tratamiento de diálisis en los siete días anteriores y presentaba mal estado general, por lo cual consultó en urgencias.

**Figura 2:**
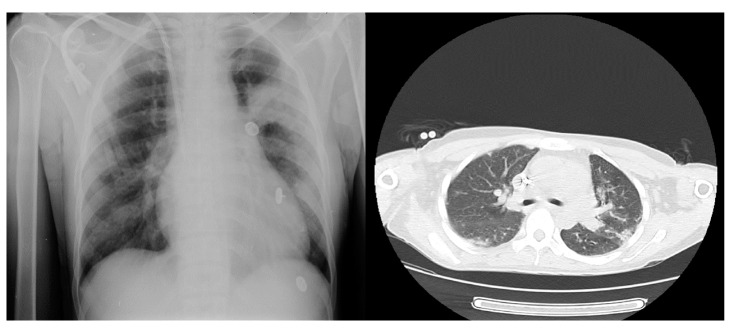
Radiografía y tomografia computarizada de tórax en el caso 2

En el examen físico se encontró presión arterial de 140/90 mm Hg, frecuencia cardiaca de 96 latidos por minuto, frecuencia respiratoria de 24 respiraciones por minuto, saturación de oxígeno de 93 %, murmullo vesicular disminuido en ambos campos pulmonares, edema de grado II en extremidades inferiores y debilidad muscular proximal, pero no se evidenció asterixis ni deterioro del estado de consciencia. Presentó hiperpotasemia y ondas T picudas en el electrocardiograma, por lo que en primera instancia se le administró una solución polarizadora de dextrosa al 10 %, insulina cristalina y gluconato de calcio y, posteriormente, diálisis durante cuatro horas, con una ultrafiltración de 1.000 ml, filtro de 170, bomba de sangre a 250 ml/min, y flujo de dializado de 500 ml/min. Se la hospitalizó y se indicó la continuación de la diálisis en días intercalados con los mismos parámetros en aislamiento respiratorio y 3 l/ min de oxígeno suplementario. Se inició el tratamiento antibiótico con 500 mg intravenosos de claritromicina cada 12 horas, 1 g intravenoso de cefepime cada 12 horas y 1 g intravenoso de vancomicina cada 48 horas después de la diálisis. La radiografía de tórax reveló aumento de la trama vascular bilateral, con patrón intersticial en la base derecha y TC de tórax con imagen en vidrio esmerilado bilateral (figura 2). Se tomó muestra con hisopado nasofaríngeo para la detección de SARS-CoV-2, la cual resultó positiva.

Los resultados de los exámenes de laboratorio durante la hospitalización se reportan en el cuadro 2. La evolución clínica fue satisfactoria y se completó un esquema de siete días de antibióticos. Se le dio el alta médica indicándose la continuidad de la diálisis en su centro de referencia.

**Cuadro 2:** Exámenes de laboratorio durante la hospitalización en piso y en la unidad de cuidados intensivos en el caso 2

Examen de laboratorio	Día 1	Día 2	Día 4	Día 6
Leucocitos (número por mm^3^)	5.700	6.200	3.100	4.800
Neutrófilos (número por mm^3^)	3.700	3.400	1.600	3.500
Linfocitos (número por mm^3^)	1.500	2000	900	900
Hemoglobina (g/dl)	11,3	11,1	11,5	12,3
Plaquetas (número por mm^3^)	91.000	85.000	94.000	94.000
Velocidad de sedimentación (mm/h)	25	NR	NR	NR
Tiempo de trombina (s)	NR	10,2	14,5	11
Tiempo parcial de tromboplastina (s)	NR	37	46,3	26,8
INR	NR	0,9	1,3	1
Creatinina (mg/dl)	19,3	19,4	8,2	11,1
BUN (mg/dl)	91	94	26	41
Urea (mg/dl)	194	201	55	87,7
Sodio (mEq/L)	145	146	134	135
Potasio (mEq/L)	7,2	7,4	4,4	49
Magnesio (mg/dl)	NR	2,4	NR	NR
Calcio (mg/dl)	NR	NR	8,2	NR
Cloro (mEq/L)	113	115	101	102
GOT (UI/L)	32	35	46	42
GPT (UI/L)	61	56	48	42
Bilirrubina total (mg/dl)	0,5	0,6	0,5	0,5
Bilirrubina directa (mg/dl)	0	0	0	0
Bilirrubina indirecta (mg/dl)	0	0	0,2	0,1
Fosfatasa alcalina (U/L)	NR	NR	192	NR
Deshidrogenasa láctica (U/L)	212	285	295	NR
PCR (mg/dl)	4,3	NR	NR	NR
Ferritina (µg/L)	211	277	455	472
Troponina I (ng/ml)	21,0	NR	0,029	0,023
Dímero D (ng/ml)	NR	NR	4.120	3.980
Panel respiratorio: influenza A, influenza B y H1N1 2009	Negativo	NR	NR	NR
Hemocultivo	Negativo	NR	NR	NR

NR: no reportado: INR: *International Normalized Ratio;* BUN: *Blood Urea Nitrogen;* GOT: *Glutamic-Oxaloacetic Transaminas*e; GPT: *Glutamic-Pyruvic Transaminase;* LDH: *Lactate Dehydrogenase*

### Caso 3

Mujer de 44 años, residente en Galapa (Atlántico) con enfermedad renal crónica permanente e hipertensión, en tratamiento de hemodiálisis tres veces a la semana desde el 2007 en sesiones de cuatro horas cada una mediante catéter permanente, y con diuresis residual. La COVID-19 se inició el 13 de junio de 2020 con fiebre no cuantificada, tos seca y disnea, por lo que fue remitida desde su unidad renal. Entre sus antecedentes refirió tabaquismo durante más de 20 años.

En el examen físico de ingreso tenía presión arterial de 150/90 mm Hg, frecuencia cardiaca de 120 latidos por minuto, temperatura de 38,7 °C, frecuencia respiratoria de 28 respiraciones por minuto, saturación de oxígeno de 90 %, y crepitación en ambos campos pulmonares en la auscultación. No se evidenció edema, asterixis, deterioro del estado de consciencia, ni debilidad.

**Figura 3:**
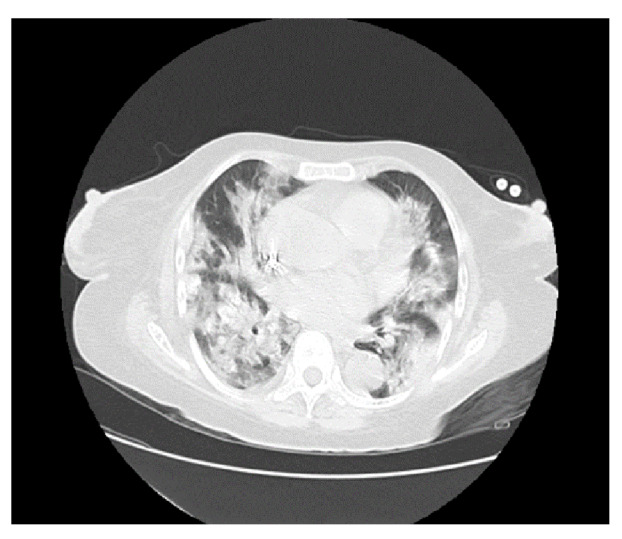
Tomografia de tórax en el caso 3

Se tomó le muestra por hisopado nasofaríngeo para SARS-CoV-2 y resultó positiva. Los resultados de los exámenes de laboratorio durante la hospitalización se presentan en el cuadro 3. En la tomografía de tórax se evidenciaron infiltrados bilaterales con patrón de vidrio esmerilado (figura 3).

**Cuadro 3:** Exámenes de laboratorio durante la hospitalización en el caso 3

Examen de laboratorio	Día 1	Día 3	Día 5
Leucocitos (número por mm^3^)	5.400	3.300	3.300
Neutrófilos (número por mm^3^)	4.200	2.300	2.500
Linfocitos (número por mm^3^)	900	400	400
Hemoglobina (g/dl)	13	12,1	12,8
Plaquetas (número por mm^3^)	277.000	316.000	384.000
Velocidad de sedimentación (mm/h)	36	NR	NR
Tiempo de trombina (s)	11	NR	12,3
Tiempo parcial de tromboplastina (s)	47,3	NR	33,2
INR	1	NR	1,1
Creatinina (mg/dl)	7,3	6	2,8
BUN (mg/dl)	67	79	41
Urea (mg/dl)	83,46	169,06	87,74
Sodio (mEq/L)	142	136	138
Potasio (mEq/L)	6,3	8,2	5,1
Magnesio (mg/dl)	2,1	2,3	1,9
Calcio (mg/dl)	9,4	8,5	NR
Cloro (mEq/L)	103	103	104
GOT (UI/L)	32	33	24
GPT (UI/L)	8	30,1	14
Bilirrubina total (mg/dl)	NR	1,2	0,6
Bilirrubina directa (mg/dl)	NR	0	0
Bilirrubina indirecta (mg/dl)	NR	0,2	0,2
Fosfatasa alcalina (U/L)	NR	128	289
Deshidrogenasa láctica (U/L)	451	NR	274
PCR (mg/dl)	33,1	NR	NR
Ferritina (µg/L)	2.880	NR	NR
Dímero D (ng/ml)	3.160	NR	NR
Troponina I (ng/ml)	0,025	0,012	NR
Panel respiratorio: influenza A, influenza B y H1N1 2009	Negativo	NR	NR
Hemocultivo	Negativo	NR	NR

NR: no reportado: INR: *International Normalized Ratio;* BUN: *Blood Urea Nitrogen;* GOT: *Glutamic-Oxaloacetic Transaminase;* GPT: *Glutamic-Pyruvic Transaminase;* LDH: *Lactate Dehydrogenase*

Se la puso en aislamiento respiratorio con oxígeno suplementario por cánula nasal en dosis de 3 l/min y se retomó la hemodiálisis en intervalos de un día y sesiones de cuatro horas cada una, con 2.500 ml de ultrafiltrado, filtro de 170, bomba de sangre a 250 ml/min y flujo de dializado de 500 ml/ min; se le administraron 40 mg de metilprednisolona intravenosa cada 12 horas, piperacilina tazobactam en dosis de 2,25 g intravenosos cada 8 horas, 500 mg intravenosos de claritromicina cada 12 horas, 500 mg de acetaminofén por vía oral cada 8 horas y 600 mg de N-acetilcisteína por vía oral cada 8 horas durante siete días. La paciente tuvo una adecuada respuesta con mejoría de los parámetros respiratorios, por lo que se le dio el alta con indicación de continuar la diálisis en su centro de referencia.

### Caso 4

Hombre de 57 años con enfermedad renal crónica permanente, hipertensión y diabetes mellitus tipo 2, en tratamiento de hemodiálisis trisemanal en sesiones de cuatro horas por sesión desde el 1° de diciembre de 2017 utilizando fístula arteriovenosa, y sin diuresis residual. La COVID-19 se inició el 27 de mayo de 2020 con fiebre de 38,8 °C, acompañada de anosmia y ageusia. A partir del 2 de junio de 2020 presentó disnea en reposo, por lo que fue remitida con reporte de PCR-RT positiva para SARS-CoV-2.

En el examen físico la presión arterial era de 128/76 mm Hg, la frecuencia cardiaca de 92 latidos por minuto, la frecuencia respiratoria de 26 respiraciones por minuto, saturación de oxígeno de 92 %, y temperatura de 38,5 °C. Se evidenció un mal patrón respiratorio, con presencia de crepitación en ambos campos pulmonares. No presentaba edema, asterixis, deterioro del estado de consciencia, ni debilidad. Dados estos hallazgos se inició la administración de 3 l/min de oxígeno y fue trasladada a la unidad de cuidados intensivos. En el cuadro 4 se reportan los resultados de los exámenes de laboratorio durante su hospitalización.

**Cuadro 4:** Exámenes de laboratorio durante la hospitalización en piso y en la unidad de cuidados intensivos en el caso 4

Examen de laboratorio	Día 1	Día 3	Día 4	Día 5	Día 6
Leucocitos (número por mm^3^)	5.300	6.900	7.100	6.000	7.900
Neutrófilos (número por mm^3^)	4.400	5.800	5.800	5.100	7.300
Linfocitos (número por mm^3^)	600	700	1.000	600	300
Hemoglobina (g/dl)	11,8	11,4	10,7	9,9	10,4
Plaquetas (número por mm^3^)	141.000	128.000	131.000	132.000	163.000
Velocidad de sedimentación (mm/h)	25	NR	NR	NR	NR
Tiempo de trombina (s)	11,5	11,3	11	12,4	13,1
Tiempo parcial de tromboplastina (s)	46,6	27,1	40,1	47,7	47
INR	1	1	1	1,1	1,2
Creatinina (mg/dl)	10,8	9,5	10,9	10,6	9,9
BUN (mg/dl)	51	49	62	68	58
Urea (mg/dl)	109	104	132	145	124
Sodio (mEq/L)	131	141	138	139	140
Potasio (mEq/L)	4,6	4,3	4,3	3,6	4,2
Cloro (mEq/L)	89	94	94	97	96
GOT (UI/L)	62	76	76	58	46
GPT (UI/L)	38	38	41	45	39
Bilirrubina total (mg/dl)	0,7	0,8	0,8	1,5	1,4
Bilirrubina directa (mg/dl)	0	0	0	0,08	0,2
Bilirrubina indirecta (mg/dl)	0	0,1	0,1	0,2	0,2
Deshidrogenasa láctica (U/L)	485	663	653	440	NR
PCR (mg/dl)	12	NR	NR	NR	NR
Ferritina (µg/L)	695	922	873	867	705
Troponina I	NR	0,153	0,160	0,073	0,08
Dímero D (ng/ml)	350	3.708	2.680	4.240	4.260

NR: no reportado: INR: *International Normalized Ratio;* BUN: *Blood Urea Nitrogen;* GOT: *Glutamic-Oxaloacetic Transaminase;* GPT: *Glutamic-Pyruvic Transaminase;* LDH: *Lactate Dehydrogenase*

**Figura 4:**
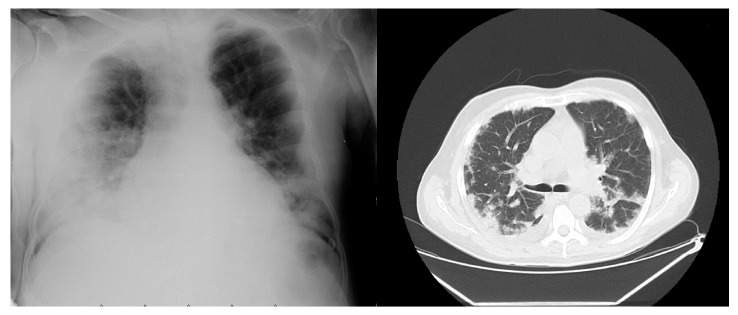
Radiografía y tomografia computarizada de tórax en el caso 4

Se le tomó una radiografía de tórax que mostró consolidación alveolar e intersticial, con redistribución de flujo, y una tomografía de tórax que mostró un patrón reticular de vidrio esmerilado basal bilateral y hacia los segmentos laterales de ambos pulmones con múltiples lesiones hiperdensas de tipo nodular (figura 4).

Se continuó la hemodiálisis en días intercalados y sesiones de tres horas cada una, con ultrafiltración de 2.000 ml, filtro de 170, bomba de sangre a 250 ml/min, y flujo de dializado de 500 ml/min; se le administraron 400 mg intravenosos de manejó moxifloxacino diarios, 500 mg de claritromicina por vía oral cada 12 horas y 600 mg intravenosos de N-acetilcisteína cada 12 horas, durante siete días, con lo cual evolucionó satisfactoriamente con mejoría del patrón respiratorio, por lo que fue trasladado a la habitación y después de una vigilancia estricta durante 48 horas se le dio el alta médica con indicaciones de continuar la diálisis en su centro de referencia.

### Caso 5

Hombre de 63 años residente en Maicao (La Guajira), con enfermedad renal crónica permanente e hipertensión y en tratamiento de hemodiálisis trisemanal desde el 2013 utilizando fístula arteriovenosa, y sin diuresis residual. La COVID-19 se inició el 14 de mayo de 2020 con fiebre y dolor lumbar que irradiaba al abdomen y los miembros inferiores. Se le internó en la unidad de cuidados intensivos de su localidad por neumonía adquirida en la comunidad e infección del torrente circulatorio; el hemocultivo fue positivo para *Staphylococcus aureus* y una resonancia magnética de columna lumbar evidenció inflamación y absceso epidural. Con ese cuadro fue remitido a este hospital de mayor complejidad. Entre sus antecedentes refirió alergia a las penicilinas.

**Figura 5:**
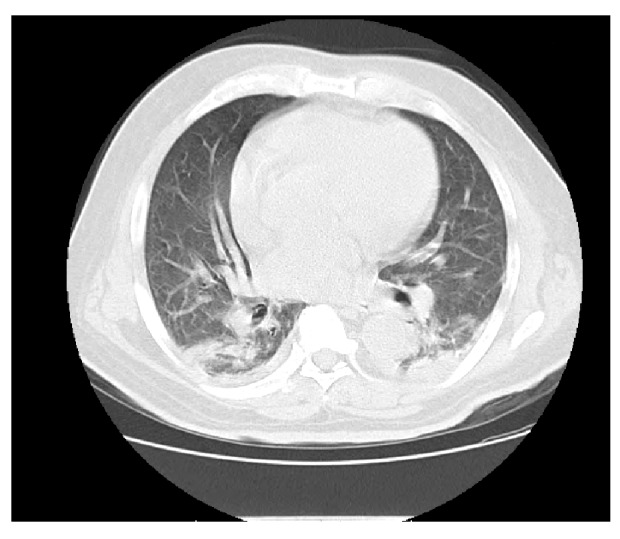
Tomografía de tórax en el caso 5

En el examen físico de ingreso se encontró al paciente en mal estado general, con facies álgida, obesidad de grado II, presión arterial de 130/70 mm Hg, frecuencia cardiaca de 110 latidos por minutos, frecuencia respiratoria de 34 respiraciones por minuto, y saturación de oxígeno de 93 %. No se evidenciaron edema, asterixis, deterioro del estado de consciencia, ni debilidad. Teniendo en cuenta el antecedente de neumonía adquirida en la comunidad, se le hizo una tomografía de tórax que evidenció ocupación alveolar del tipo de neumonía y compromiso intersticial (figura 5). Se le tomó la muestra para la PCR-RT de detección del SARS-CoV-2, la cual fue positiva. Los resultados de los exámenes de laboratorio durante la hospitalización se reportan en el cuadro 5.

**Cuadro:** 5. Exámenes de laboratorio durante la hospitalización en el caso 5

Examen de laboratório	Día 1	Día 3
Leucócitos (número por mm^3^)	18.300	9.600
Neutrófilos (número por mm^3^)	15.900	6.900
Linfocitos (número por mm^3^)	1.400	1.700
Hemoglobina (g/dl)	9,3	7,8
Plaquetas (número por mm^3^)	364.000	329.000
Tiempo de trombina (s)	11,1	NR
Tiempo parcial de tromboplastina (s)	19,6	NR
INR	1	NR
Creatinina (mg/dl)	8,3	8,4
BUN (mg/dl)	64	54
Urea (mg/dl)	136	115
Sodio (mEq/L)	152	149
Potasio (mEq/L)	5,4	5
Cloro (mEq/L)	110	114
GOT (UI/L)	63	28
GPT (UI/L)	58	25
Bilirrubina total (mg/dl)	0,9	NR
Bilirrubina directa (mg/dl)	0	NR
Bilirrubina indirecta (mg/dl)	0,1	NR
Fosfatasa alcalina (U/L)	113	NR
Deshidrogenasa láctica (U/L)	419	NR
PCR (mg/dl)	9	NR
Ferritina (µg/L)	1.290	1.280
Troponina I	0,494	NR
Hemocultivo	Negativo	NR

NR: no reportado: INR: *International Normalized Ratio;* BUN: *Blood Urea Nitrogen;* GOT: *Glutamic-Oxaloacetic Transaminase;* GPT: *Glutamic-Pyruvic Transaminase;* LDH: *Lactate Dehydrogenase*

Se le indicó retomar la diálisis en días intercalados con ultrafiltración de 3.000 ml, filtro de 170, bomba de sangre a 250 ml/min, y flujo de dializado de 500 ml/min. Se le administraron 500 mg intravenosos diarios de meropenem y 500 mg intravenosos de vancomicina cada 48 horas después de la diálisis, y 30 mg intravenosos de meperidina cada seis horas, y oxígeno suplementario a 3 l/min, con lo cual hubo mejoría clínica. El paciente se encontraba casi a 350 km de su domicilio y familia y al quinto día de hospitalización solicitó el alta voluntaria. Se le indicó continuar con la diálisis y con los antibióticos en su centro de referencia.

### Caso 6

Mujer de 59 años, residente en Barranquilla (Atlántico), con enfermedad renal crónica permanente e hipertensión, diabetes mellitus tipo II con requerimiento de insulina, y obesidad. Recibía hemodiálisis trisemanal en sesiones de 4 horas mediante fístula arteriovenosa desde el 28 de abril de 2017, sin diuresis residual. La COVID-19 apareció el 10 de junio de 2020 con episodios eméticos, picos febriles no cuantificados, astenia, adinamia, tos no productiva y disnea de medianos esfuerzos. En el ingreso refirió haber debido asistir a diálisis cuatro días antes, pero no asistió a la sesión. También manifestó haber discontinuado la insulina y la medicación antihipertensiva 24 horas antes de la consulta.

**Figura 6:**
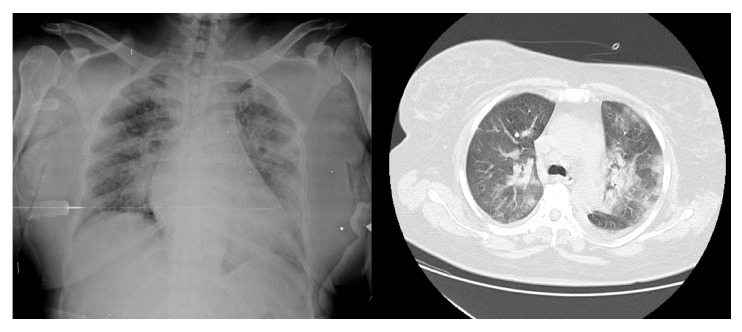
Radiografía y tomografia computarizada de tórax en el caso 6

En el examen físico de ingreso su presión arterial fue de 150/90 mm Hg, la frecuencia cardiaca de 109 latidos por minuto, la temperatura de 37 °C, la frecuencia respiratoria de 24 respiraciones por minuto, la saturación de oxígeno de 88 %, una disminución del murmullo vesicular con crepitación fina en ambas bases, pero de predominio izquierdo, roncos generalizados y edemas de grado III en miembros inferiores. No se evidenció asterixis, deterioro del estado de consciencia, ni debilidad. Los resultados de los exámenes de laboratorio se reportan en el cuadro 6. En la radiografía de tórax se evidenció un patrón reticular difuso con áreas de compromiso alveolar en el lóbulo medio y en la base del pulmón izquierdo, y en la tomografía de tórax, un patrón retículo-alveolar parahiliar bilateral, con imágenes del tipo de vidrio esmerilado (figura 6). Se le tomó muestra con hisopado nasofaríngeo para detectar el SARS-CoV-2 mediante PCR-RT y esta fue positiva.

**Cuadro 6:** Exámenes de laboratorio durante la hospitalización en el caso 6

Examen de laboratorio	Día 1	Día 4	Día 5
Leucocitos (número por mm^3^)	8.700	7.500	6.700
Neutrófilos (número por mm^3^)	7.200	6.100	5.600
Linfocitos (número por mm^3^)	1.000	900	700
Hemoglobina (g/dl)	13,2	10,2	9,2
Plaquetas (número por mm^3^)	276.000	318.000	324.000
Velocidad de sedimentación (mm/h)	28	NR	NR
Tiempo de trombina (s)	10,8	NR	13,2
Tiempo parcial de tromboplastina (s)	32,9	NR	49,4
INR	1	NR	1,2
Creatinina (mg/dl)	12,7	6,6	8,6
BUN (mg/dl)	55	32	53
Urea (mg/dl)	117,7	68,48	113,42
Sodio (mEq/L)	144	140	140
Potasio (mEq/L)	6,3	4,5	4,7
Magnesio mg/dl	2,1	1,8	NR
Calcio (mEq/L)	NR	8	8,2
Cloro (mEq/L)	103	100	100
GOT UI/L	40	NR	55
GPT UI/L	20	NR	22
Bilirrubina total mg/dl	NR	NR	1,3
Bilirrubina directa mg/dl	NR	NR	0
Bilirrubina indirecta mg/dl	NR	NR	0,5
Fosfatasa alcalina U/L	97	NR	NR
Deshidrogenasa láctica U/L	495	NR	41
PCR mg/dl	22,7	NR	NR
Ferritina Ug/L	1.990	NR	NR
Dímero D ng/ml	196,1	NR	NR
Troponina I ng/ml	<0,240	NR	NR
Panel respiratorio: influenza A, influenza B y H1N1 2009	Negativo	NR	NR

NR: no reportado: INR: *International Normalized Ratio;* BUN: *Blood Urea Nitrogen;* GOT: *Glutamic-Oxaloacetic Transaminase;* GPT: *Glutamic-Pyruvic Transaminase;* LDH: *Lactate Dehydrogenase*

La paciente fue hospitalizada en aislamiento respiratorio con indicación de continuar la hemodiálisis en días intercalados en sesiones de cuatro horas cada vez, con 2.000 ml de ultrafiltración, filtro de 170, bomba de sangre a 250 ml/min, y flujo de dializado de 500 ml/min. Se le aplicó tratamiento antibiótico con piperacilinatazobactam en dosis de 2,25 g intravenosos cada 8 horas, 500 mg intravenosos de claritromicina cada 12 horas, y 40 mg intravenosos de metilprednisolona cada 12 horas. Durante su hospitalización se observó mejoría y al noveno día de tratamiento se le dio el alta médica con recomendaciones estrictas e indicación de continuar la diálisis en su centro de referencia.

### Caso 7

Hombre de 57 años, residente en Barranquilla (Atlántico) con enfermedad renal crónica permanente e hipertensión, diabetes mellitus y cardiopatía isquémica, sometido a hemodiálisis trisemanal desde enero de 2016 mediante fístula arteriovenosa y sin diuresis residual; ingresó el 5 de junio de 2020 por fiebre de 38 °C de predominio nocturno asociada a astenia y adinamia. No había asistido a su hemodiálisis en los últimos 4 días.

**Figura 7:**
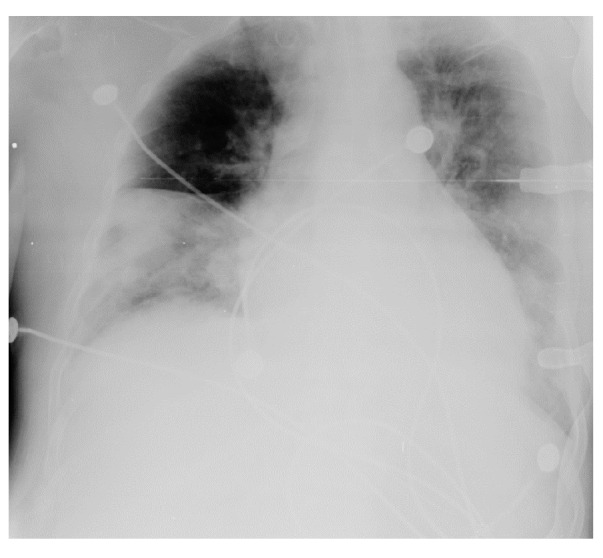
Radiografía de tórax en el caso 7

En el examen físico de ingreso el paciente presentó mal estado general, somnolencia, presión arterial de 127/65 mm Hg, frecuencia cardiaca de 105 latidos por minuto, frecuencia respiratoria de 24 respiraciones por minuto, temperatura de 37,7°C, saturación de oxígeno de 82 % con fracción inspirada de oxígeno (FIO_2_) de 21 %, ruidos cardiacos rítmicos, murmullo vesicular disminuido, con crepitación en bases bilaterales, y presencia de fístula arteriovenosa funcional en miembro superior izquierdo. No se evidenció edema. Los resultados de los exámenes de laboratorio en el momento de ingreso y durante la hospitalización se presentan en el cuadro 7. En la radiografía de tórax se evidenciaron infiltrados alveolares bibasales (figura 7). Se le dieron 3 l/min de suplencia de oxígeno por cánula nasal, pero la evolución fue desfavorable: empeoramiento de la hipoxemia y alteración de la dinámica respiratoria, por lo que se le hizo intubación orotraqueal y fue trasladado a la unidad de cuidados intensivo como caso sospechoso de COVID-19. Se le inició la hemodiálisis y el tratamiento con 500 mg intravenosos de claritromicina cada 12 horas, 1 g intravenoso de cefepime cada 12 horas, así como N-acetilcisteína e insulina.

**Cuadro 7:** Exámenes de laboratorio durante la hospitalización en el caso 9

Examen de laboratorio	Día 1	Día 2	Día 3	Día 4	Día 5
Leucocitos (número por mm^3^)	9.300	17.100	11.700	13.000	8.200
Neutrófilos (número por mm^3^)	7.900	15.900	10.179	10.920	7.380
Linfocitos (número por mm^3^)	855	427	842	832	492
Hemoglobina (g/dl)	9,3	10,5	10	11	12,4
Plaquetas (número por mm^3^)	133.000	162.000	160.000	181.000	121.000
Velocidad de sedimentación (mm/h)	43	NR	NR	NR	NR
Tiempo de trombina (s)	12,2	12,6	12,5	16,7	15,3
Tiempo parcial de tromboplastina (s)	51,3	44,8	56,7	>60	>60
INR	1,1	1,1	1,1	1,5	1,4
Glicemia (mg/dl)	NR	218	91	NR	225
Creatinina (mg/dl)	18,1	11,9	12,3	NR	9
BUN (mg/dl)	51	56	62	40	49
Urea (mg/dl)	109,14	119,84	132,68	85	104,86
Sodio (mEq/l)	139	138	139	165	150
Potasio (mEq/l)	4,5	5,4	5	4,7	3,8
Calcio (mEq/l)	NR	NR	NR	8,9	8,6
Cloro (mEq/l)	93	94	96	120	110
GOT (UI/L)	15	33	38	70	122
GPT (UI/L)	10	14	12	15	15
Bilirrubina total (mg/dl)	0,9	1	1	1,4	1,7
Bilirrubina directa (mg/dl)	0	0	0	0,038	0,172
Bilirrubina indirecta (mg/dl)	0,1	0	0	0,3	0,3
Deshidrogenasa láctica (U/L)	378	NR	390	537	658
PCR mg/dl	28,4	NR	NR	NR	NR
Ferritina (Ug/L)	NR	3.650	3.490	4.030	7.010
Dimero D (ng/ml)	2.788	441,3	440	720	6.180
Troponina I (ng/ml)	0,314	0,958	1,95	NR	0,894
Panel respiratorio: influenza A, influenza B y H1N1 2009	NR	Negativo	NR	NR	NR
Gases arteriales, pH			7,36	7,44	7,43
pO_2_			49	54	57
pCO_2_			41	41	41
Lactato	NR	NR	1,3	3,2	1,8
HCO_3_			23,3	27,8	27,2
Exceso de base			-2,2	3,6	2,9
Pa/FiO_2_			49	54	71

NR: no reportado: INR: *International Normalized Ratio;* BUN: *Blood Urea Nitrogen;* GOT: *Glutamic-Oxaloacetic Transaminase;* GPT: *Glutamic-Pyruvic Transaminase;* LDH: *Lactate Dehydrogenase*

Se mantuvo la hemodiálisis en días intercalados con sesiones de tres horas, ultrafiltración de 2.000 ml, filtro de 170, bomba de sangre a 250 ml/ min, y flujo de dializado a 500 ml/min. El paciente presentaba inestabilidad hemodinámica, por lo que se inició el soporte vasopresor y el tratamiento con 45 mg intravenosos de metilprednisolona cada 12 horas. Posteriormente se lo trató con hemodiafiltración. El paciente presentó síndrome de dificultad respiratoria grave, choque y bradicardia extrema con posterior asistolia, por lo que se le hicieron las maniobras de reanimación, pero no recuperó los signos vitales. Finalmente falleció.

## Discusión

En el 2019 se reportaron en el departamento del Atlántico 2.600 pacientes dependientes de algún tipo de tratamiento de reemplazo renal, 1.620 de ellos en Barranquilla ^12^. Durante el pico de la pandemia de COVID-19 en el país (junio de 2020), cuando el mayor número de casos reportados se registraba precisamente en esta ciudad, se hospitalizaron en el Departamento de Nefrologia de la Clínica de la Costa siete pacientes con enfermedad renal crónica en hemodiálisis a quienes se les comprobó la presencia de infección por el SARS-CoV-2, cuyas características clínicas, resultados de exámenes de laboratorio e imágenes diagnósticas se resumen en el cuadro 8.

**Cuadro 8:** Resumen de los casos clínicos reportados

Características clínicas	Caso 1	Caso 2	Caso 3	Caso 4	Caso 5	Caso 6	Caso 7
Edad (años)	43	43	44	57	63	59	57
Sexo	Hombre	Mujer	Mujer	Hombre	Hombre	Mujer	Hombre
Antecedentes de contacto con caso	No	No	No	No	No	No	No
positivo de COVID-19							
Días que suspendió el tratamiento de	7	5	0	0	0	4	0
remplazo renal							
Diabetes mellitus	No	No	No	Sí	No	Sí	Sí
Hipertensión arterial	Sí	Sí	Sí	Sí	Sí	Sí	Sí
Fiebre	Sí	Sí	Sí	Sí	No	Sí	Sí
Tos	Sí	Sí	Sí	No	No	Sí	No
Disnea	No	Sí	Sí	Sí	No	Sí	Sí
Astenia o adinamia	Sí	No	No	No	No	Sí	Sí
Diarrea	No	Sí	No	No	No	No	No
Vómitos	No	No	No	No	No	Sí	No
Anosmia	Sí	No	No	Sí	No	No	No
Ageusia	Sí	No	No	Sí	No	No	No
**Datos de exámenes de laboratorio**							
Leucocitosis	No	No	No	No	Si	No	Si
Leucopenia	Sí	Sí	No	No	No	No	No
Neutrofilia	Sí	No	No	Sí	Sí	Sí	Sí
Neutropenia	No	Sí	No	No	No	No	No
Linfocitosis	No	No	No	No	No	No	No
Linfopenia	Sí	Sí	Sí	Sí	No	Sí	Sí
Elevación de marcadores hepáticos	Sí	Sí	No	Sí	Sí	Sí	Sí
Prolongación de tiempos de coagulación	No	No	No	No	No	No	Sí
Elevación de dímero D	NR	Sí	Sí	Sí	NR	No	Sí
Elevación de ferritina	NR	Sí	Sí	Sí	Sí	Sí	Sí
Elevación de LDH	Sí	Sí	Sí	Sí	Sí	Sí	Sí
Elevación de troponina I	NR	Sí	No	Sí	Sí	Sí	Sí
Elevación de proteína C reactiva	Sí	Sí	Sí	Sí	Sí	Sí	Sí
**Imágenes diagnósticas**							
Patrón alveolar intersticial	Sí	Sí	No	Sí	Sí	Sí	Sí
Patrón en vidrio esmerilado	Sí	Sí	Sí	Sí	No	Sí	No

LDH: *Lactate Dehydrogenase*

En Colombia, especialmente en la región Caribe y en Barranquilla, coexisten la desigualdad social y la pobreza. En este contexto, los pacientes en diálisis tienen mayor vulnerabilidad sanitaria durante la etapa de pandemia, pues deben recorrer largas distancias y hacer uso del transporte público para acceder a su tratamiento. Además, el número de pacientes por unidad de diálisis es elevado, por lo que deben ser ubicados uno muy cerca de otro durante el tratamiento; además, estas unidades son espacios cerrados, lo que conlleva un alto riesgo de contraer la infección por SARS-CoV-2 ^17^^,^^18^, y sus condiciones de pobreza y alfabetización limitan el cumplimiento del aislamiento y el distanciamiento social ^19^.

Los síntomas más frecuentes que presentaron los pacientes de los casos descritos en el presente artículo fueron fiebre, tos y disnea, lo que concuerda con lo reportado en la literatura. Asimismo, se estableció la presencia de anosmia, ageusia, diarrea y vómito en algunos casos menos comunes, lo que también coincide con las publicaciones disponibles ^20^^,^^21^, y específicamente en pacientes con COVID-19 en hemodiálisis ^22^^,^^23^.

Por otra parte, en los exámenes de laboratorio de la mayoría de los pacientes se encontró linfopenia, la cual se ha asociado con la uremia debido al deterioro de la función de los linfocitos y los granulocitos, lo que conlleva una respuesta inmunitaria alterada frente a la infección por SARS-CoV-2 ^24^. También asociada con la linfopenia, se ha descrito una mayor gravedad de la enfermedad ^25^^,^^26^. En todos los pacientes que se presentan aquí hubo alteración en los marcadores de inflamación, como el dímero D, la proteína C reactiva y la ferritina, los cuales se consideran de mal pronóstico por su asociación con la tormenta de citocinas y el consecuente estado proinflamatorio sostenido ^27^^,^^28^, y la mayoría de ellos presentó elevación de los biomarcadores hepáticos previamente asociados con el mal pronóstico de la COVID-19 ^29^.

En cuanto a los hallazgos radiológicos, en la mayoría de los casos se encontró un patrón alveolar intersticial e imágenes en vidrio esmerilado, siendo estas las clásicas descripciones asociadas con la infección por SARS-CoV-2 ^30^^,^^31^ según la clasificación CO-RADS 6, es decir, hallazgos sospechosos de COVID-19 con prueba de PCR positiva ^32^.

En cuanto al diagnóstico, dado que en nuestro medio no se hace rutinariamente la PCR-RT para la detección del SARS-CoV-2, no se pudo determinar con certeza el inicio de la infección activa en los pacientes reportados. En el momento de su ingreso se les hizo la prueba diagnóstica, la cual fue positiva en los siete casos ^33^.

Los pacientes en hemodiálisis crónica se consideran un grupo de alto riesgo para complicaciones graves de la COVID-19 debido a su estado de inmunosupresión y la presencia de comorbilidades ^24^^,^^34^. A pesar de esto, en la mayoría de nuestros pacientes la evolución clínica fue satisfactoria, lo que es atribuible al diagnóstico y el tratamiento oportunos, y solo falleció un paciente con múltiples comorbilidades que aumentan el riesgo de mortalidad ^35^.

En vista de la elevada mortalidad de la COVID-19, en muchos países los ministerios de salud han establecido consensos y guías de manejo ^36^ que incluyen medidas de prevención, información y educación de los pacientes, la búsqueda de casos en las unidades renales, el aislamiento de los pacientes sospechosos o confirmados de infección activa por SARS-CoV-2, el uso del equipo de protección personal y la desinfección de superficies y equipos médicos ^37^. En Colombia los resultados del consenso se implementaron partir de junio de 2020 ^38^. Además, dado que la propagación en este grupo de pacientes es más elevada, llegando a cifras del 16 %, se sugiere que, una vez detectados los casos, se los traslade oportunamente a centros hospitalarios para tratar la enfermedad aguda y evitar la propagación de la infección en la unidad renal ^39^^,^^40^.

## Conclusión

Hay suficiente evidencia de que los pacientes en hemodiálisis crónica tienen una mayor propensión a infectarse con el SARS-CoV-2, pero se han presentado datos controversiales sobre la mortalidad. En algunas series publicadas, la mortalidad ha alcanzado el 30,5 % ^41^, en tanto que en el Registro COVID-19 de la Sociedad Española de Nefrología, la mortalidad global es del 23 % ^42^, principalmente en los pacientes en hemodiálisis crónica con mayor edad, tratamientos de hemodiálisis más prolongados y desarrollo frecuente de neumonía. Según estas cifras, de cada cuatro pacientes en diálisis positivos para el coronavirus, uno no sobrevive. Sin embargo, otras publicaciones refieren una menor mortalidad global en pacientes en hemodiálisis comparados con la población general, lo que se atribuye a la anticoagulación propia del tratamiento, al deterioro de la función celular inmunitaria y la incapacidad de presentar tormenta de citocinas ^43^^,^^44^.

Lo que no se debe olvidar es que, debido a sus propias características y a las de su particular tratamiento crónico, estos pacientes necesitan una protección especial por su propia vulnerabilidad y por la cercanía de sus terapeutas, sobre todo ante la posibilidad de contactos cercanos con "portadores sanos" Este inconveniente debe superarse estableciendo protocolos de detección precoz para así aislar a los pacientes afectados y evitar posibles brotes en las unidades de diálisis que difícilmente podrían controlarse y tendrían un efecto indeseable en el personal sanitario y en los pacientes.

Dada la incertidumbre que todavía existe sobre la prevención, el diagnóstico y el manejo de estos casos, es urgente que en todas las unidades renales se adopten los protocolos de trabajo generados por los ministerios de salud de los países que se revisan mediante consensos basados en los conocimientos aportados por las experiencias clínicas y la investigación para responder a los desafíos a los que nos enfrenta esta pandemia.

Colombia ha establecido recomendaciones sistemáticas de trabajo para las unidades renales con el objeto de proteger la salud de todos los pacientes que asisten a ellas y del personal de salud, incluidos los médicos nefrólogos tratantes ^45^; entre ellas se destacan las siguientes medidas: hacer la prueba de PCR y la rápida a todos los pacientes de las unidades de diálisis y al personal para establecer los cercos epidemiológicos, extremar el aislamiento y separar a los pacientes positivos de los negativos durante su desplazamiento y la hemodiálisis, contemplando la eventual hospitalización de los casos positivos en centros adecuadas para tal fin, así como espaciando las sesiones de hemodiálisis en los casos sospechosos hasta obtener el resultado de la PCR.

Es imperativo suministrar elementos de protección personal completos e idóneos al equipo de salud para no tener que implementar aislamientos preventivos que hagan dificultosa la continuidad de los tratamientos por falta de profesionales capacitados para ello, así como proteger a los cuidadores, dotar de tapabocas a los pacientes e iniciar el tratamiento temprano y oportuno de los casos de los sintomáticos para reducir las complicaciones y la mortalidad.

Es necesario hacer estudios prospectivos con el mayor número posible de pacientes para entender el espectro clínico completo y lograr un diagnóstico y tratamiento adecuados de los pacientes con COVID-19 en hemodiálisis, así como establecer protocolos de vigilancia epidemiológica para controlar tempranamente los eventuales focos de contagio masivo.
